# Unsupervised Medical Image Segmentation Based on the Local Center of Mass

**DOI:** 10.1038/s41598-018-31333-5

**Published:** 2018-08-29

**Authors:** Iman Aganj, Mukesh G. Harisinghani, Ralph Weissleder, Bruce Fischl

**Affiliations:** 1 Department of Radiology, Massachusetts General Hospital, Harvard Medical School, Boston, MA USA; 2 Computer Science and Artificial Intelligence Laboratory, Massachusetts Institute of Technology, Cambridge, MA USA; 3 Center for Systems Biology, Massachusetts General Hospital, Harvard Medical School, Boston, MA USA

## Abstract

Image segmentation is a critical step in numerous medical imaging studies, which can be facilitated by automatic computational techniques. Supervised methods, although highly effective, require large training datasets of manually labeled images that are labor-intensive to produce. Unsupervised methods, on the contrary, can be used in the absence of training data to segment new images. We introduce a new approach to unsupervised image segmentation that is based on the computation of the local center of mass. We propose an efficient method to group the pixels of a one-dimensional signal, which we then use in an iterative algorithm for two- and three-dimensional image segmentation. We validate our method on a 2D X-ray image, a 3D abdominal magnetic resonance (MR) image and a dataset of 3D cardiovascular MR images.

## Introduction

Image segmentation is the process of partitioning the set of image pixels into subsets, where the pixels in each subset are related, e.g. with respect to their intensities and/or locations. Segmentation of biomedical images is a central step in many medical imaging studies. Automating segmentation can be highly beneficial, as it can enable the large-scale studies needed to find subtle changes and disease effects.

Numerous approaches to medical image segmentation have been proposed (see surveys)^1,2^. Provided that a large training dataset of a specific class of images – with ground-truth labels – is available, *supervised* segmentation can be effective in producing accurate results. Such a dataset can be exploited to train a deep neural network^3^ or create probabilistic atlases^4^, which can in turn generate accurate segmentations of new images in the same image class. Creating training datasets, however, requires manual delineation of labels by experts, which is labor-intensive and costly for a dataset that is sufficiently large for training. Furthermore, new image types are constantly being developed, making the creation of the manual training sets needed for supervised segmentation an onerous and ongoing task. Finally, in the case of an uncommon type of data, providing a large-enough dataset to label may be challenging.

*Unsupervised* segmentation, in contrast, has the advantage of not requiring training data to segment images and therefore is helpful in the absence of a manually-labeled dataset. Unsupervised segmentation methods are more generally applicable and more robust to atypical or unseen situations^5–10^. In addition, the results of such methods are potential starting points for manual segmentation, thereby expediting the creation of training datasets. Since even an unsupervised segmentation algorithm may be effective in segmenting some but not all classes of images, it is important to explore new methodologies to expand the suitable options for unsupervised segmentation of various classes of medical images.

In this work, we introduce a new approach to unsupervised medical image segmentation, which is based on the computation of the one-dimensional (1D) local center of mass (CM). We first propose an efficient method to compute, for each pixel in a 1D signal, the CM of the region containing the pixel. In other words, by using the information from the entire signal, we group the pixels based on the CM of the regions where they are located. Next, we exploit this method for 2D and 3D image segmentation, by computing the local CMs of image pixels in many different orientations and then iteratively updating the segmentation label of each pixel by choosing from the labels at its local CMs. (We use the word “pixel” interchangeably to mean the index of a sample, a pixel, or a voxel, in a discrete-domain 1D signal, a 2D image, or a 3D volume, respectively.) We evaluate our approach qualitatively on a 2D hand X-ray image and a 3D abdominal magnetic resonance (MR) image, as well as quantitatively on a 3D dataset of 10 cardiovascular MR images.

In the following, we describe the proposed method in detail (the Methods section), present experimental results (the Results section), discuss them (the Discussion section) and conclude the paper (the Conclusion section).

## Methods

### Computation of 1D Local Center of Mass

Let $f:{\rm{\Omega }}\to {\mathbb{R}}$ be a 1D discrete-domain image-intensity signal of length *N*, with ${\rm{\Omega }}:=\{1,\ldots ,N\}$. We group the pixels of *f* into disjoint regions based on the computed CM of each pixel’s putative region, which we shall refer to as the *local* CM, *C*: Ω → ${\mathbb{R}}$. In other words, the value of the local CM at *n*, *C*_*n*_, is the CM of the region containing the *n*^th^ pixel. We calculate the local CM as:

1 $${C}_{n}=\frac{{\sum }_{m=1}^{N}\,{w}_{m,n}m}{{\sum }_{m=1}^{N}\,{w}_{m,n}},$$

where the nonnegative weighting *w*_*m*,*n*_ must be computed from *f* such that it is large if the *m*^th^ and *n*^th^ pixels are in the same region and small otherwise. Consequently, for pixels that are in the same region, the function *C* will point to roughly the same location (the region’s CM), thereby assigning those pixels to the same cluster. A pixel is therefore clustered by exploiting the information provided by the entire signal, as opposed to only its neighboring pixels. Computing *C* (the entire set {*C*_*n*_|*n* ∈ Ω}) is, however, in general expensive, costing ${\mathscr{O}}({N}^{2})$.

### Computational Complexity Reduction

**Figure 1:**
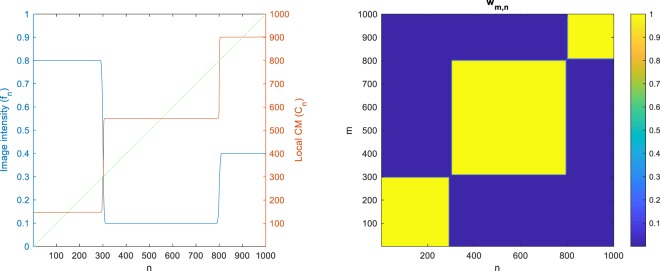
Left: Image intensity profile (*f*_*n*_, blue), local CM (*C*_*n*_, red) and the identity line (*n*, dotted green). Right: The weighting (*w*_*m*,*n*_).

To make the problem tractable, we must choose *w* such that, while it serves the abovementioned pixel-grouping purpose, the computational cost of *C* can also be reduced. Here, by choosing *w* as follows, we will significantly reduce the cost of computing *C*, to ${\mathscr{O}}(N)$:

2 $${w}_{m,n}:\,={e}^{-|{D}_{m}-{D}_{n}|},$$

where,

3 $${D}_{n}:\,=\alpha \sum _{i=1}^{n}\,{|{f}_{i+1}-{f}_{i}|}^{p},$$

with manually chosen *α*, *p* > 0. (We define ${f}_{N+1}:\,={f}_{N}$ for ease of notation.) One can see that the more and stronger the signal edges between the *m*^th^ and *n*^th^ pixels are, the larger |*D*_*m*_ − *D*_*n*_| and thus the smaller *w*_*m*,*n*_ is, indicating that the two pixels are in different regions. On the other hand, if there are few edges between the *m*^th^ and *n*^th^ pixels, *w*_*m*,*n*_ will be large, indicating that the two pixels are in the same region. Figure 1 shows an example of a 1D signal *f*_*n*_ (left, blue curve), along with its computed *w*_*m*,*n*_ (right). Note that Eq. (3) is trivially extendable to multichannel (such as RGB) images, by computing the sum separately for each channel and adding the results leading to an aggregate *D*_*n*_.

As for the computation of *C*, since *D*_*n*_ is non-decreasing with respect to *n*, *w*_*m*,*n*_ can be rewritten as ${e}^{-{D}_{n}}{e}^{{D}_{m}}$ for *m* ≤ *n* and ${e}^{{D}_{n}}{e}^{-{D}_{m}}$ for *m* > *n*, leading to the following expansion of Eq. (1) to compute the elements of *C*:

4 $${C}_{n}=\frac{{e}^{-{D}_{n}}{\sum }_{m=1}^{n}\,{e}^{{D}_{m}}m+{e}^{{D}_{n}}{\sum }_{m=n+1}^{N}\,{e}^{-{D}_{m}}m}{{e}^{-{D}_{n}}{\sum }_{m=1}^{n}\,{e}^{{D}_{m}}+{e}^{{D}_{n}}{\sum }_{m=n+1}^{N}\,{e}^{-{D}_{m}}}.$$

Given that all the sums in Eqs (3) and (4) can be pre-computed *recursively* in ${\mathscr{O}}(N)$ and stored for all *n*, the entire *C* can now be computed efficiently and non-iteratively in ${\mathscr{O}}(N)$. Figure 1 (left) shows the plot of *C*_*n*_ (red) for our example, in addition to the identity line (dotted green) for the reference. As expected, *C*_*n*_ is piecewise constant and its value points to the CM of each interval in the signal (e.g., it intersects the identity line at the centers of the intervals).

### Image Segmentation

We now employ the aforementioned 1D local CM computation method to develop an iterative algorithm for unsupervised segmentation of a 2D or 3D image with *N* pixels. Ω = {1, …, *N*} now reflects the set of pixel indices of the image. Essentially, we assign the label of an estimated local CM to pixels pointing to that CM, causing the pixels in the same region to eventually share the same label. This is reminiscent of watershed methods^5^, where pixels corresponding to a common point form a catchment basin with a unique label. However, the proposed local-CM-based approach labels a pixel using the information from the entire image, i.e. beyond just the neighboring pixels, while avoiding a quadratic computational complexity.

**Figure 2:**
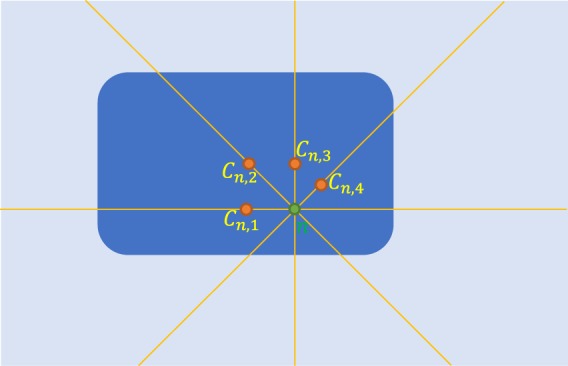
Local CMs, ${{\mathscr{C}}}_{n}=\{{C}_{n,1},\ldots ,{C}_{n,4}\}$, of the *n*^th^ pixel of the image, along *K* = 4 orientations.

We have shown in the previous subsections how to efficiently compute the local CMs from a 1D signal. For 2D and 3D segmentation, however, we must exploit the spatial relations among pixels in all dimensions. Accordingly, we make use of the proposed 1D CM computation technique and find the 1D local CMs independently in *K* orientations uniformly distributed on the unit semicircle or hemisphere (for the 2D and 3D cases, respectively). For the *n*^th^ pixel, we obtain a set of *K* pixels, ${{\mathscr{C}}}_{n}:\,=\{{C}_{n,k}\in {\rm{\Omega }}|k=1,\ldots ,K\}$, where *C*_*n*,*k*_ is (the index of) the pixel closest to the local CM of the *n*^th^ pixel along the *k*^th^ orientation (i.e., nearest-neighbor interpolation of the CM on the Cartesian grid). An example of a pixel with its local CMs computed in several orientations is illustrated in Fig. 2. Note that the discrete derivative of the image in Eq. (3) is computed along the chosen orientation. To ensure that the set ${{\mathscr{C}}}_{n}$ has a uniform spatial (rather than angular) distribution, we down-sample it nonuniformly with a rate proportional to 1/*d*_*k*_ or $1/{d}_{k}^{2}$ in the 2D and 3D cases, respectively, where *d*_*k*_ is the distance between the *n*^th^ pixel and *C*_*n*,*k*_.

For a 3D image of size *N*_1_ × *N*_2_ × *N*_3_ (where *N*_1_*N*_2_*N*_3_ = *N*), let us consider the computation of the local CMs of all pixels in the orientation along the first image dimension, i.e. *C*_*n*,*k*_ for all *n* ∈ Ω, for the fixed orientation *k* that corresponds to the *x*-axis. Using the computational complexity reduction technique described in the Computational Complexity Reduction section, local CMs of each row will cost ${\mathscr{O}}({N}_{1})$. Given that there is a total of *N*_2_*N*_3_ rows, the computation of the local CMs for all image pixels (for the fixed orientation) will cost ${\mathscr{O}}({N}_{2}{N}_{3}\cdot {N}_{1})={\mathscr{O}}(N)$. Similarly, the computational complexity is the same for any other orientation. Therefore, we pre-compute the local CMs along all *K* orientations, i.e. the entire $\{{{\mathscr{C}}}_{n}|n\in {\rm{\Omega }}\}$, efficiently in ${\mathscr{O}}(KN)$ and store it in memory.

Next, we initialize a label image, *L*^0^: Ω → ${\mathbb{N}}$, with *N* unique randomly-located labels. We then start an iterative loop, consisting of two phases, where we update the label image, *L*, at each iteration. For all *n* ∈ Ω, we reassign the label *L*_*n*_ of the *n*^th^ pixel by making use of the pre-computed set ${{\mathscr{C}}}_{n}$ (that includes the local CMs of the *n*^th^ pixel in different orientations):

- In the first phase, we randomly choose a pixel ${m}_{{\rm{rand}}}\in {{\mathscr{C}}}_{n}$ and assign its label from the last iteration to *L*_*n*_; i.e., ${L}_{n}\leftarrow {L}_{{m}_{{\rm{rand}}}}^{0}$.
- In the second phase, we instead find the most frequent label (from last iteration) corresponding to the pixels in ${{\mathscr{C}}}_{n}$ and assign it to *L*_*n*_; i.e., ${L}_{n}\leftarrow {\rm{mode}}\{{L}_{m}^{0}|m\in {{\mathscr{C}}}_{n}\}$.

The generated *L* at each iteration becomes the new *L*^0^ for the next iteration. We start with the first phase and after a predefined number of phase-1 iterations, *t*, switch to the second phase. Then, during the phase-2 iterations, we stop the loop if there is no more change in *L*, or when the iteration number reaches a predefined maximum. Finally, expecting the resulting *L* to have *l* ≪ *N* unique values, we map the labels in *L* to {1, …, *l*}, before returning *L* as the segmentation results. Note that, due to the randomness introduced in the first phase, one may obtain slightly different results by repeating a segmentation experiment.

## Results

We implemented our unsupervised segmentation algorithm in MATLAB and evaluated it on 2D and 3D medical images. We also compared it with three other unsupervised segmentation algorithms: the watershed segmentation^5^, a Gaussian-mixture-model-based hidden-Markov-random-field (GMM-HMRF) model^7^ initialized with k-means clustering and the simple linear iterative clustering (SLIC) superpixel algorithm^8^ (with constant compactness). We used the MATLAB Image Processing Toolbox™ for watershed and SLIC and a public MATLAB toolbox^7^ for GMM-HMRF. For watershed segmentation, we created the edge map of the image using the Sobel filter, computed its *h-minima* transform, i.e. removed its local minima that were shallower than the scalar *h* and then applied the watershed transform. For GMM-HMRF, we set the maximum number of iterations to the suggested value of 10 for both the EM algorithm and the MAP estimation.

After initially trying the values of *p* = 1, 2, 3 for the *p*-norm in Eq. (3), we heuristically concluded that our method produced the most reasonable results with p^*^ = 2. For each image, we ran our method with ranges of values for *α* (the Computational Complexity Reduction section) and for the number of phase-1 iterations *t* (the Image Segmentation section), the watershed algorithm with a range of values for *h*, the GMM-HMRF algorithm with ranges of values for the number of regions *r* (for both the GMM-HMRF and its k-means initializer) and the number of GMM components *g* and the SLIC algorithm with a range of values for the number of superpixels *s*. Next, for each method, we determined the optimal parameter values: in the 2D X-ray Image Segmentation and the 3D Abdominal MR Image Segmentation sections by heuristically choosing the least over-segmented result among those segmentation results that reflected all major anatomical regions and in the 3D Cardiovascular MR Image Segmentation section once by choosing the result with the highest mean Dice score and a second time by cross validation.

Label edges are shown in black for better contrast and clearer visualization in the figures. We also used a simple greedy algorithm to roughly match the colors of overlapping labels among subfigures for easier comparison.

### 2D X-ray Image Segmentation

**Figure 3:**
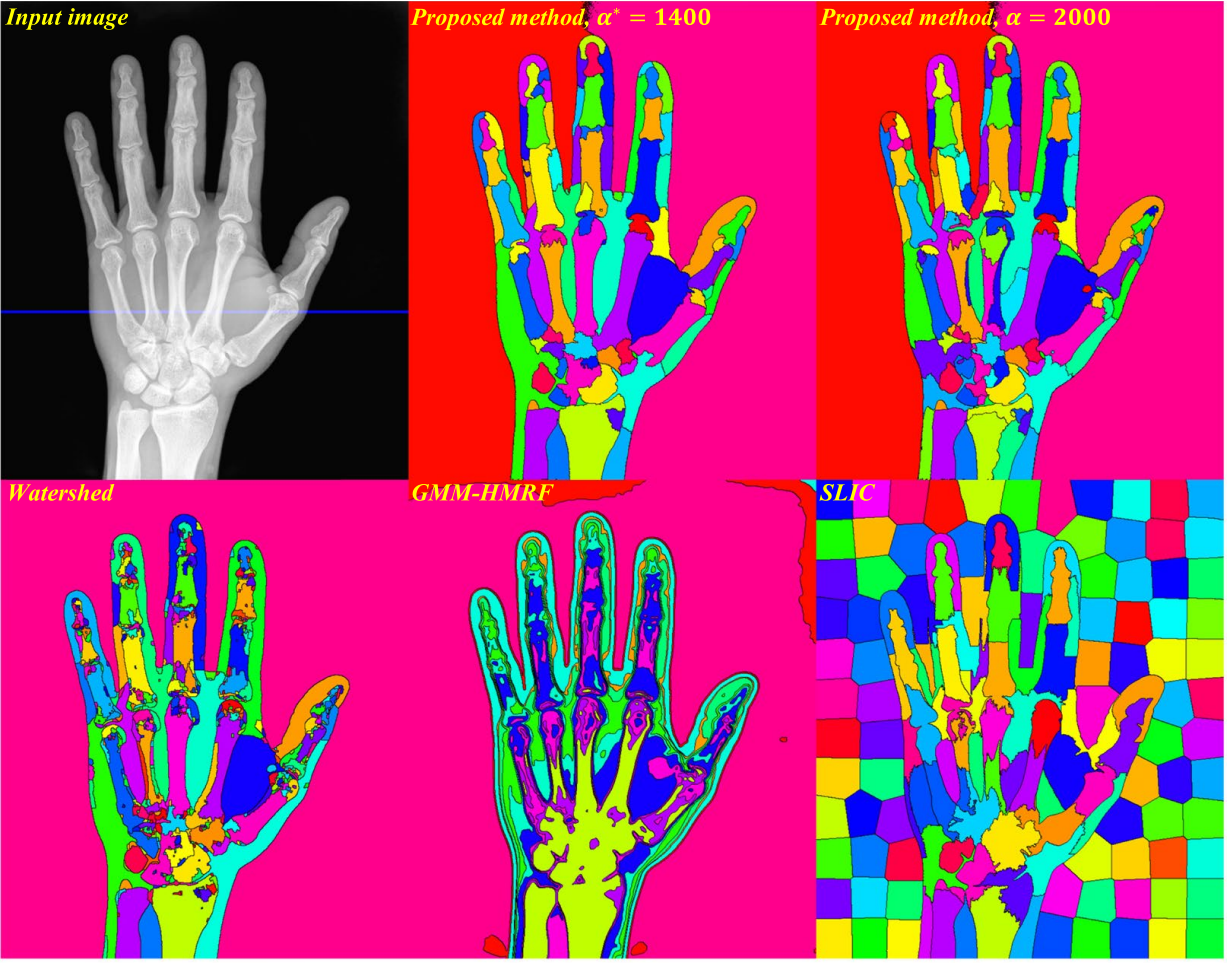
The input X-ray image (top, left), with segmentation results using the proposed local-CM-based (top, middle and right), the watershed (bottom, left), the GMM-HMRF (bottom, middle) and the SLIC (bottom, right) methods (X-Ray image courtesy of Trace Meek).

We first evaluated the four (proposed local-CM-based, watershed, GMM-HMRF and SLIC) methods on a publicly available 2D hand X-ray image^11^, with the dimensions of 750 × 880 pixels (Fig. 3, top left). Here and in the 3D Abdominal MR Image Segmentation section, images were initially normalized to have intensity values between 0 and 1. We chose an angular resolution of 1° and computed the local CMs in *K* = 180 orientations uniformly distributed on the semicircle. Figure 3 (top) shows segmentation results by our local-CM-based method, with optimal t^*^ = 2000 phase-1 iterations (10,000 total iterations) and optimal α^*^ = 1400 (middle) and *α* = 2000 (right). Figure 3 (bottom) depicts the results for the watershed method with optimal h^*^ = 0.116 (left), the GMM-HMRF model with optimal r^*^ = 11 and g^*^ = 2 (middle) and the SLIC algorithm with optimal s^*^ = 130 (right).

**Figure 4:**
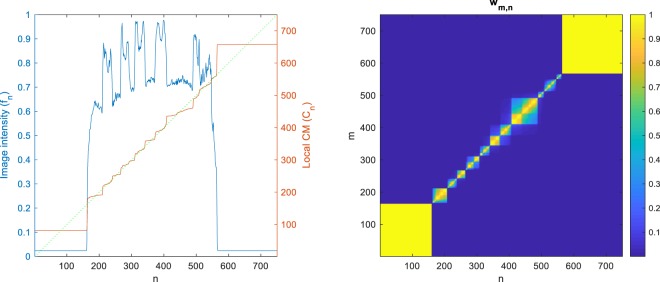
Left: Image intensity profile (blue) of the horizontal blue line in Fig. 3 (top, left), local CM (red) and the identity line (dotted green). Right: The weighting (*w*_*m*,*n*_).

The 1D image intensity profile along the horizontal blue line in Fig. 3 (top, left) is plotted in Fig. 4 (blue curve), along with the local CM of this 1D signal computed from Eq. (4) with *α* = 200 (red curve) and the identity function for the reference (green dotted line). The local CM curve (red) can be seen to be almost piecewise constant, with its values indicating the centers of the 1D intervals.

### 3D Abdominal MR Image Segmentation

Next, we applied the four methods to a 3D abdominal MR image acquired as part of a previous study^12^; quoting from which: “All participants were 18 y of age or older. Informed consent was obtained after the nature and possible consequences of the studies were explained. […] The Joslin Diabetes Center Committee on Human Studies and the Massachusetts General Hospital Institutional Review Board approved the protocols. FDA Investigational New Drug approval was obtained for the use of ferumoxytol for MRI.”

**Figure 5:**
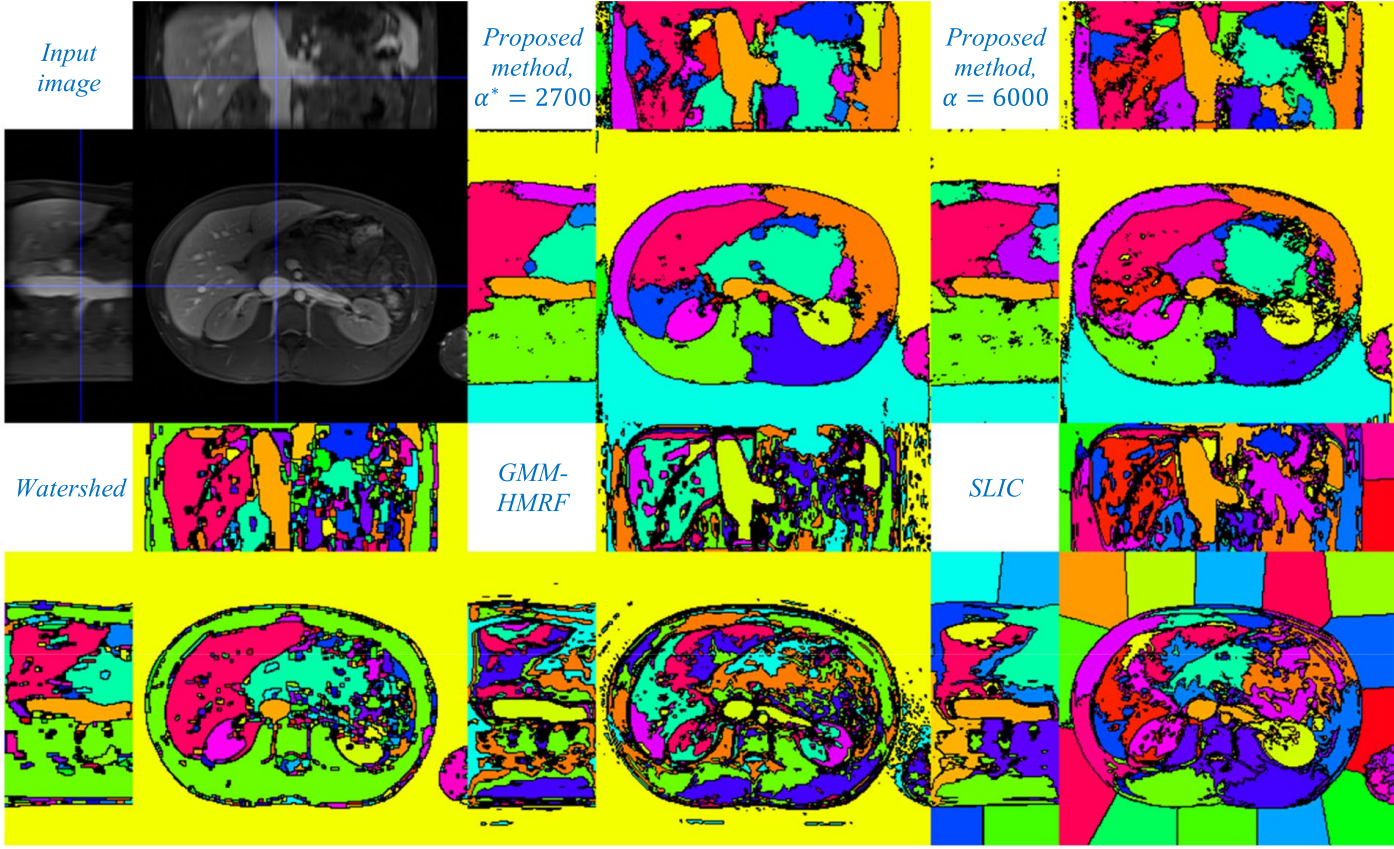
Input abdominal MR image (top, left), with segmentation results using the proposed local-CM-based (top, middle and right), the watershed (bottom, left), the GMM-HMRF (bottom, middle) and the SLIC (bottom, right) methods.

The T1-weighted image had been acquired with the voxel size of 1.4 × 1.4 × 3.5 mm³, after the administration of a magnetic nanoparticle agent. We upsampled the volume in the slice-selection direction with linear interpolation to obtain a (1.4 mm)³ isotropic-voxel volume of the size 224 × 256 × 98 voxels. Figure 5 illustrates coronal, sagittal and axial slices of the image (top, left), along with those of the 3D segmentation results using the four methods. We chose an angular resolution of 10° and computed the local CMs in *K* = 193 orientations uniformly distributed on the hemisphere. For the proposed method, we show the results obtained with optimal α^*^ = 2700 and *α* = 6000 and optimal t^*^ = 1500 phase-1 iterations (5000 total iterations). Optimal parameter values for other methods were h^*^ = 0.154 (watershed), r^*^ = 9 and g^*^ = 1 (GMM-HMRF) and s^*^ = 46 (SLIC).

### 3D Cardiovascular MR Image Segmentation

Lastly, for a quantitative assessment of the performance of the four methods, we used a public dataset of 10 cardiovascular MR images^13^ acquired at 1.5 T, which also included manual segmentation of the ventricular myocardium and the blood pool. We used the available cropped images (“axial, cropped training data”) with varying sizes ranging from 97 × 102 × 164 to 165 × 239 × 181 voxels and the voxel size of 0.9 × 0.9 × 0.85 mm³. We divided each image by its intensity standard deviation and then normalized the images by the same constant so their maxima were on average 1 (their minima were 0). We chose an angular resolution of 20°, resulting in *K* = 54 uniformly distributed orientations on the hemisphere and ran our algorithm for 5000 iterations. To segment each image, we used a range of values for each parameter of each method (see Table 1). For each of the two manual labels, we identified the corresponding label in each resulting segmentation as the one with the maximal Dice’s overlap coefficient with the manual label. Then, for each subject and each method, we chose the optimal segmentation result that produced the maximal Dice score (averaged across the two labels).

**Table 1:** Summary of the quantitative results on cardiovascular MR images.

	Proposed	Watershed	GMM-HMRF	SLIC
Optimal Dice	**0.60 ± 0.02**	0.53 ± 0.04	0.55 ± 0.01	0.56 ± 0.01
CV Dice	0.51 ± 0.04	0.41 ± 0.05	**0.54 ± 0.01**	0.49 ± 0.02
Optimal parameters	α^*^ = 2100 ± 300 t^*^ = 3050 ± 400	h^*^ = 0.41 ± 0.05	r^*^ = 4 ± 0.5 g^*^ = 1 ± 0	s^*^ = 8 ± 2
Tested range of parameters	*α*: 100~8000 *t*: 50~5000	*h*: 0~1.5	*r*: 2 ~ 20 *g*: 1 ~ 5	*s*: 2 ~ 500
CPU Runtime	37000 ± 11000 s (10 ± 3 hr)	6 ± 1 s	9000 ± 2000 s (2.5 ± 0.6 hr)	**2.5 ± 0.4 s**

**Figure 6:**
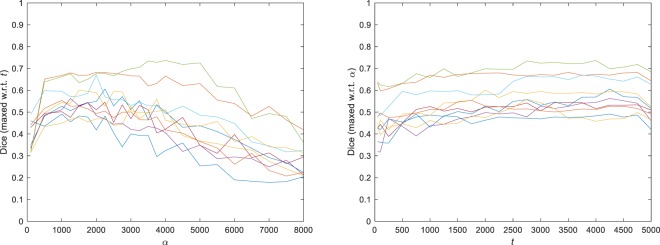
Dice scores for all 10 subjects, for a fixed *α* maximized over *t* (left) and for a fixed *t* maximized over *α* (right).

Cross-subject mean (±standard error) of the optimal Dice scores and optimal parameters are shown in Table 1. As can be seen, the proposed local-CM-based method resulted in a higher mean optimal Dice score than the competing methods did. Figure 6 illustrates, for all the 10 subjects, how robust the mean Dice score was with respect to varying *α* (left) and *t* (right). The vertical axis shows the best score achieved with respect to the remaining parameter. Alternatively, we also performed a leave-one-out cross validation (CV) by testing the methods on a left-out subject with the median of the optimal parameter values computed from the other 9 subjects, repeating it 10 times (each time leaving out a different subject) and averaging the Dice scores. As Table 1 shows, the proposed method achieved a higher CV Dice score than the watershed and SLIC methods did, but a lower one than the GMM-HMRF method did.

We distributed the above experiments to different processors with inhomogeneous hardware. Hence, to compare the runtime of the methods on the same hardware, we reran the optimal experiments (two experiments at a time) on a desktop PC with two Intel®Xeon® E5-2637 v3 3.50 GHz processors, each with four cores. We did not parallelize our codes; however, MATLAB may multithread some of its internal functions. The cross-subject means of the runtimes are shown in Table 1. Due to the complexity of the mode function, the bulk of our prototype’s runtime is spent in phase-2 iterations, with a phase-2 iteration being on average 7 times slower than a phase-1 iteration. Note that, to ensure convergence for all tests, we ran our method for a high total of 5000 iterations; however, one could reduce the number of phase-2 iterations and still obtain reasonable results. Further actions to gain speed would be to use a smaller number of orientations, *K* and to run the algorithm on a graphics processing unit (GPU).

## Discussion

As the figures demonstrate, the proposed local-CM-based method generally produced less over-segmented results than the rest of the methods did (except in the background). This may be because our method assigns each pixel label by considering – not only the neighboring pixels, but – the entire image. Increasing *h* improved the over-segmentation by the watershed method, but at the price of not capturing some boundaries. The watershed method, however, resulted in better-defined borders between articular cartilages (Fig. 3) and a single-segment liver (Fig. 5). The GMM-HMRF model seems to emphasize more the image intensity than the geometry of a segment, hence leading to rather tissue- than organ-specific segmentation. This model is not so successful in segmenting individual bones (Fig. 3) and some organs (Fig. 5), possibly because it assigns a single gaussian to each identified connected tissue type. Regarding the SLIC segmentation, the borders of its individual bone and organ labels do not seem as well-defined as the segmentation by the proposed method. Also, as expected, larger regions such as the background are divided into many superpixels. To accurately evaluate the performance of the segmentation methods, we have avoided any postprocessing, such as region-merging techniques^6^ that could potentially alleviate the over-segmentation issue.

In our quantitative validation (the 3D Cardiovascular MR Image Segmentation section), the proposed local-CM-based method achieved the highest optimal Dice overlap score among the four methods, but came in second place with regard to the CV Dice score, after the GMM-HMRF model. The latter may be because the ventricular myocardium and the blood pool are rather tissue types, whose image intensities are suitable for mixture models (see above). Note that the Dice scores were produced without any shape prior or training. Nonetheless, given that we tuned one or two parameters for each method, our *evaluation* of the four methods can be considered as mostly but not totally unsupervised. We have empirically computed the optimal parameter values in our experiments and provided them throughout the Results section. In practice, these values can be a good starting point for the interested reader who wants to try the proposed CM-based segmentation on a new image (that has intensities normalized between 0 and 1). Automatic optimization of the parameters of our method (*α* and *t*) is part of our future research.

The often-improved results of the proposed method came at the price of a higher computational cost (Table 1). To reduce the runtime, the algorithm can be run with considerably lower numbers of orientations (*K*) and iterations, while still producing reasonable results. Moreover, our publicly available codes (the Data Availability section) are GPU-compatible, making it possible to significantly speed up the computation.

Our algorithm uses the CM of a region as a reference point, whose label it iteratively propagates to the rest of the pixels in that region. If such a reference point is located inside the region, it will be unique to the region. For an interval in a 1D signal (which is always convex), the CM is trivially located inside the region (Figs 1 and 4). This is, yet, not guaranteed to be the case in higher dimensions, unless the region is convex. That is why we did not use the direct definition of the 2D/3D CM (with double/triple sums); instead, we exploited 1D CMs in different orientations to approximate local 2D/3D CMs. Being always inside the region, 1D CMs can be used as reference points with labels that are unique to the region. A drawback of this approximation though is that, in a highly nonconvex region, more iterations may be needed for the label to propagate entirely. In some cases, the algorithm may even converge to a locally optimal result where a non-convex region is over-segmented. The two labels computed for the background in Fig. 3 (top) and for the liver in Fig. 5 (top) are examples of such a phenomenon. Nevertheless, choosing a large enough number of phase-1 iterations (*t*) can alleviate this issue and, in fact, many nonconvex regions can be seen to have been successfully segmented by the proposed method.

Lastly, if an initial segmentation for some parts of the image is available, it can be used to initialize the labels (instead of random assignment). The labels in those regions can be chosen to either remain fixed or get updated in the iterations. This is especially useful for semi-automatic segmentation, where the user provides some information to the algorithm on which pixels should be grouped together.

## Conclusion

We have introduced a new unsupervised medical image segmentation approach that groups the pixels in each region based on the *local CMs* of the region. We proposed an efficient scheme for the 1D case, in ${\mathscr{O}}(N)$, which we extended to higher dimensions via an iterative algorithm. Through qualitative and quantitative validation, we have shown the proposed method to often outperform three existing unsupervised segmentation methods. Automatic optimization of the two parameters of the method, *α* and the number of phase-1 iterations *t*, is part of the future work.

## Data Availability

Our MATLAB codes for the proposed method are publicly available at: www.nitrc.org/projects/seg. The hand X-ray image^11^ (https://cnx.org/contents/FPtK1zmh@9.1:vaCcDeiY@4/Medical-Imaging) in the 2D X-ray Image Segmentation section and the de-identified cardiovascular MR images^13^ (http://segchd.csail.mit.edu) in the 3D Cardiovascular MR Image Segmentation section are also publicly available. The abdominal MR image in the 3D Abdominal MR Image Segmentation section was secondarily used from a different study^12^ and – at least for now – is not available to the public due to institutional policies.
